# Mini-Review: Gut-Microbiota and the Sex-Bias in Autoimmunity – Lessons Learnt From Animal Models

**DOI:** 10.3389/fmed.2022.910561

**Published:** 2022-06-16

**Authors:** Elizabeth C. Rosser, Nina M. de Gruijter, Diana E. Matei

**Affiliations:** ^1^Centre for Adolescent Rheumatology Versus Arthritis at University College London (UCL), University College London Hospital (UCLH) and Great Ormond Street Hospital (GOSH), London, United Kingdom; ^2^Centre for Rheumatology Research, Division of Medicine, University College London, London, United Kingdom; ^3^Division of Infection and Immunity, Institute of Immunity and Transplantation, University College London, London, United Kingdom

**Keywords:** gut-microbiota, inflammation, autoimmunity, sex, immune system

## Abstract

It is well appreciated that there is a female preponderance in the development of most autoimmune diseases. Thought to be due to a complex interplay between sex chromosome complement and sex-hormones, however, the exact mechanisms underlying this sex-bias remain unknown. In recent years, there has been a focus on understanding the central pathogenic role of the bacteria that live in the gut, or the gut-microbiota, in the development of autoimmunity. In this review, we discuss evidence from animal models demonstrating that the gut-microbiota is sexually dimorphic, that there is a bidirectional relationship between the production of sex-hormones and the gut-microbiota, and that this sexual dimorphism within the gut-microbiota may influence the sex-bias observed in autoimmune disease development. Collectively, these data underline the importance of considering sex as a variable when investigating biological pathways that contribute to autoimmune disease risk.

## Introduction

One of the strongest risk factors for developing autoimmunity is female sex (1). Although the mechanistic reasons underlying the strong female sex-bias in autoimmune conditions are unclear, it is likely to be strongly influenced by sex-differences in immune system function. Generally, innate and adaptive immune responses are stronger in females than males. Classic examples include heightened interferon type 1 production by activated female plasmacytoid dendritic cells (pDCs) (2) and stronger humoral immune responses in females, with higher antibody titres at baseline and in response to vaccination (3).

Sex determinants such as sex-chromosomes and sex-hormones influence differences in immune system function. Immune system-related genes are encoded on the X and Y chromosomes (4), and sex steroid hormones such as testosterone, oestrogens and progesterone directly impact immune cells by binding to intracellular and extracellular sex hormone receptors (5). However, this is not the whole picture. Sex influences a wide variety of host responses, which could have indirect effects on the immune system. This is supported by evidence demonstrating that the sex-bias in some experimental models of autoimmunity is sensitive to environmental factors. For example, differences in disease risk or severity are less pronounced in certain housing conditions or in germ-free mice (6). These data suggest that the commensal organisms that colonize barrier surfaces–more commonly known as microbiota–may directly impact sex-bias-associated autoimmune disease risk.

The term microbiota refers to the collection of micro-organisms that share our body space, the greatest number of which are located in the gastro-intestinal tract (7). Recently, it has become evident that pathological changes to the gut-microbiota, or dysbiosis, play a central role in influencing the aberrant immune responses that contribute to autoimmune development (8). Animal models have been pivotal in demonstrating this association. For example, K/BxN mice, which develop a spontaneous erosive arthritis, and SKG mice, which develop a severe spondyloarthropathy, do not develop arthritis when housed in germ-free conditions or when treated with broad spectrum antibiotics (9, 10). Treatment with oral antibiotics suppresses disease in a wide variety of inducible models of autoimmunity, ranging from arthritis models (11) to models of multiple sclerosis (MS) and uveitis (12–15). Multiple studies have also demonstrated that changes to the gut-microbiota are associated with the progression of autoimmune models including collagen-induced arthritis (16) and systemic lupus erythematosus (SLE) (17). This review will summarize recent research suggesting that sex influences the gut-microbiota and that sex-hormones directly impact the gut-microbiota, which in turn influences the production of sex-hormones. These data highlight how two risk factors influencing autoimmunity, sex and dysbiosis, communicate and how animal research can give insights into these biological processes. We also discuss evidence from specific experimental models where sexual dimorphism in the gut-microbiota impacts autoimmune disease development. The purpose of this review is to accentuate the diverse effects sex can have on host physiology, demonstrating the importance of reporting sex-dependent effects by including both sexes in animal research.

## Differences in Gut-Microbiota of Male and Female Mice

There are differences in the gut-microbiota in male and female mice (a summary of sex-associated bacterial species highlighted in referenced studies is summarized in Table 1). In C57BL/6–the most common strain used in animal research−17 operational taxonomic units (OTU) are more abundant in male versus female mice (e.g., *Lachnospiraceae, Clostridium, Ruminoccoceae* and *Allobaculum*), and 11 OTU are more abundant in females (e.g., *Bacteroidetes* and *Barnesiella*) (18). However, in B6.129S wild-type mice *Peptococacceae* and *Streptococacceae* are more abundant in male mice, while *Turicibacter* and *Clostridiaceae* are more abundant in females (19). As these contrasting studies were carried out in different animal facilities using different analysis techniques (for example different sequencing depths) it is hard to untangle whether these differences are driven by strain background or subtle differences in housing conditions. In a study comparing C57BL/6 mice and BALB/c mice housed in the same animal facility, strain- and sex-dependent effects on the gut-microbiota remained (20). *Lactobacillus plantarum, Bacteroides distasoni, Clostridium* spp. and Turicibacter were enriched in C57BL/6 females compared to males, whilst *Bifidobacterium* was enriched in BALB/c females compared to males. Interestingly, sex-differences in the gut-microbiota correlated with the expression of several genes associated with immune system function in the intestinal tissue. The abundance of female-enriched bacteria species such as *Clostridium leptum* positively correlated with *IL-2rb, Ccr3*, and *Cd80* expression in female C57BL/6 mice, and between male-enriched bacterial species such as *Faecalibacterium prausnitzii* and *Clostridium ramosum* positively correlated with and *Apoe, IL-1*β and *Stat4* expression in male BALB/c mice (20).

**Table 1:** Bacterial genus/species driving post-pubescent sex differences in referenced studies.

**References**	**Strain**	**Health status**	**Highlighted bacterial genus/species driving sex differences**	**Reported difference between sexes**	**Effect of microbiome differences on the host**
Bridgewater et al. (18)	C57BL/6	Naïve	*Allobaculum, Bifidobacterium, Clostridium* XIVa	Increased in males	Males were found to be resistant to the effects of stress on activity whereas females showed decreased locomotion after stress.
			*Barnesiella, Porphyromonadaceae*	Increased in females	
Kozik et al. (19)	B6.129S	Naïve	*Peptococcaceae, Streptoccocaceae*	Increased in males	Males developed more severe colitis
			*Turicibacter, Clostridiaceae*	Increased in females	
Elderman et al. (20)	C57BL/6	Naïve	*Eggerthela, Allobaculum* (not significantly)	Increased in males	Bacteria increased in abundance in females associated with increased activation, proliferation and migration of leukocytes
			Clostridium difficile, *Clostridium leptum, Enterococcus, Turicibacter*	Increased in females	
	BALB/c	Naïve	*Eggerthela, Bifidobacterium*	Increased in males	Bacteria increased in abundance in males associated with proliferation of lymphocytes, T cells in particular and migration of leukocytes
			*Prevotella* spp., *Turicibacter* (not significantly)	Increased in females	
Org et al. (21)	C57BL/6	Naïve	*Coprococcus, Bacteroides*	Increased in females	N/A
	C3H/He	Naïve	*Akkermansia, Coprobacillus, Ruminococcus, Suterella*	Increased in males	N/A
Bolnick et al. (22)	C57BL/6	High-fed diet	*Lactobacillus, Alistipes, Clostridium*, and *Lachnospiraceae*	Increased in males	N/A
		High-fed diet	*Lactobacillus, Alistipes, Clostridium*, and *Lachnospiraceae*	Decreased in females	
Bridgewater et al. (18)	C57BL/6	High-fed diet	*Ruminococcacea*	Increased in males	N/A
		High-fed diet	*Lachnospiraceae, Ruminococcacea, Peptococcaceae*	Increased in females	
Wang et al. (23)	C57BL/6	Naïve, colonized with human microbiota	*Parabacteroides distasonis, Blautia faecis*	Increased in males	N/A
			*Clostridium* groups, *Escherichia fergusonii, Shigella sonnei*	Increased in females	
Fransen et al. (24)	C57BL/6	Naïve	*Ruminococcaceae* and *Rikenellaceae*	Increased in males	Male microbiota upregulates DNA repair and cell cycle genes in female recipients. Female microbiota upregulated IL-10 signaling and completement system genes, influenced by regulation of type I interferon (IFN) production in male recipients.
			*Desulfovibrionaceae, Lactobacillaceae*	Increased in females	
Zhang et al. (17)	MRL/lpr	Model of SLE	*Lachnospiraceae*	Increased in females	The increased abundance of lachnospiraceae may influence disease development
			*Bifidobacterium*	Decreased in females	
Yurkovetskiy et al. (6)	NOD	Model of Type 1 Diabetes	Experiment 1: *Porphyromonadceae, Kineospariaceae, Veillonellaceae*	Increased in males	Post-pubescent females develop worse disease than post-pubescent males
			Experiment 2: *Enterobacteriaceae, Peptococcaceae*	Increased in males	
			Experiment 3: *Lactobacillaceae, Cytophagaceae*	Increased in males	
			Experiment 4: *Peptostreptococcaceae, Bacteroidaceae*	Increased in males	
Markle et al. (25)	NOD	Model of Type 1 Diabetes	*Roseburia, Coprococcus, Bilophilia*	Increased in males	Female mice develop worse disease than males, colonization with male microbiota protects females from disease
			Lachno I.S, Parabacteroides	Increased in females	
			Rosburia, *Blautia, Coprococcus*	Increased in females colonized with male microbiota	
			*Peptococcus*	Decreased in females colonized with a male microbiota	
Gomez et al. (26)	HLA-DRB1*0402	Arthritis-resistant control mice	*Bifidobacterium pseudolongum* subsp. *Globosum, Parabacteroides distasonis*	Increased in males	Sex-differences are lost in arthritis-susceptible HLA-DRB1*0401 mice
			*Barnesiella viscericola*	Increased in females	

Larger scale studies—one comparing sex-differences in the gut-microbiota in 8 strains from cross-collaborative mouse resource, and one independent analysis of 89 inbred mouse strains (21, 27)—demonstrate the impact of strain background, and therefore genotype, on sex-associated differences in gut-microbiota composition (21, 27). In the study comparing 89 inbred strains there were clear differences between the sexes in every strain, but the largest sex-differences were seen in C57BL/6J (females-enriched for *Coprococcus*, and males-enriched for *Bacteroides)* and C3H/HeJ mice (males-enriched for *Akkermansia, Coprobacillus, Ruminococcus, Suterella*). When the entire population analysis was interrogated together, the magnitude and direction of changes were driven by an interplay between sex and genotype (21).

Sex-dependent differences in the gut-microbiota are also impacted by diet with a high-fat diet (HFD)-fed leading to sexually divergent effects on the gut-microbiota. HFD in male mice increases the abundance of *Lactobacillus, Alistipes, Clostridium*, and *Lachnospiraceae*, whilst a HFD in female mice reduces these strains (22). In an in-depth study by Bridgewater and colleagues, sex-differences were observed in C57BL/6J mice fed standard chow, but in mice fed HFD the sex-dependent shifts were more pronounced (18). In this study, the authors did not observe opposite shifts in the bacterial species of high-fat diet fed male and female mice, but rather differences in the relative abundance of certain clades (18). More specifically, although 10 OTUs shifted in the same direction in both females and males in response to HFD (either increased or decreased in both), 31 OTUs were only affected in females and 22 OTUs were only affected in males by HFD.

Data from male and female germ-free C57BL/6J mice colonized with the same “human” microbiota (taken from one donor fed a vegetarian, high inulin diet) suggest that sex influences the ability to accommodate intestinal bacterial species (23). Despite being colonized with bacteria from the same human donor, colonized germ-free mice still displayed sex-differences in the gut-microbiota. 13 OTUs were higher in males, including *Parabacteroides distasonis* and *Blautia faecis*, whilst 33 OTUs were higher in females including *Clostridium* groups, *Escherichia fergusonii* and *Shigella sonnei* (23). Although the exact mechanisms are yet to be elucidated, these sex-difference in the ability to accommodate different bacteria is likely due to microbiota-independent sex-differences in the intestinal immune system. For example, in the study by Fransen et al. (24), sex-differences in the expression of interferon type 1 genes are present in the intestines of uncolonised mice. The authors hypothesized that lower expression of interferon type 1 genes in male mice support the colonization of bacterial groups *Alistipes, Rikenella*, and *Porphyromonadaceae*, which were overrepresented in the male microbiota versus female mice (24). These bacterial species, in turn, were associated with inflammation and DNA damage when transferred to females. Thus, microbiota-independent sexual dimorphism in the immune system might lead to the selection of a sex-specific microbiota, which then drives further divergence in immune response between males and females.

## Bidirectional Relationship Between Sex-Hormones and Gut-Microbiota

How does sex shape the gut-microbiota? As many sex-differences in the gut-microbiota are altered by sexual maturation in mice and are modified by the surgical removal of reproductive organs *via* gonadectomy, sex-hormones probably play a dominant role. In NOD mice, which have well-documented sex-differences in the gut-microbiota, there are no sex-differences in the gut-microbiota prior to puberty (6). In male NOD mice, there is a pronounced shift in the gut-microbiota post-pubertally compared to pre-pubertal animals. In female NOD mice, puberty has limited effects on the gut-microbiota, suggesting a dominant effect of testosterone on the gut-microbiota in this model (6). To address this directly, the authors gonadectomised male mice, which shifted the gut-microbiota toward a female gut-microbiota profile compared to sham-operated male mice (6). Elegantly, to eliminate the impact of specific pathogen free (SPF) housing conditions on these observations, the authors colonized male and female germ-free NOD mice with female microbiota, finding similar differences between colonized germ-free male and female NOD mice post-pubertally (6).

Gonadectomy was also shown to impact sexual dimorphism within the gut-microbiota in other studies. However, underlining the complex interplay between diet, genotype and the gut-microbiota–the effects of gonadectomy on the gut-microbiota were different depending on strain and diet (21). Overall, gonadectomy had a greater impact on the gut-microbiota of males fed standard chow and females fed a high-fat diet (21). Testosterone treatment of male gonadectomised mice could prevent the effects of gonadectomy on the gut-microbiota of C57BL/6 and C3H/HeJ mice, but not of DBA/2J mice (21). Although this study is confounded by the fact that different strains were housed in different animal facilities, these data form the most direct evidence that sex-hormones alter the composition of the gut-microbiota.

The relationship between sex-hormones and the gut-microbiota is likely to be bidirectional. Stool levels of estradiol, progesterone and corticosterone are reduced in germ-free C57BL/6 mice compared to SPF mice (28). Male germ-free NOD mice have lower levels of testosterone (25), while female germ-free NOD mice have higher levels of testosterone compared to their colonized counterparts (25). Colonization of NOD mice with microbiota containing over-represented male-associated bacterial species modulates the levels of sex-hormones in circulation (25). Following fecal transplant in microbiota-depleted mice fed broad-spectrum antibiotics, the levels of testosterone in the donor mouse can be predicted by the gut-microbiome in the recipient mouse (29). Certain bacterial species, such as those belonging to the *Actinobacteria, Proteobacteria*, and *Firmicutes* phyla, can metabolize steroid hormones through the expression of enzymes such as hydroxysteroid dehydrogenase (HSD) (30). Furthermore, disrupting the microbiota through antibiotics treatment reduces the levels of steroids within the intestine (31). More directly, intestinal micro-organisms from humans regulate testosterone levels through reversible 17β reduction of androgens by HSD (32). These data suggest that the components of the gut-microbiome can directly regulate the levels of sex-hormones, and particularly testosterone, which is an important consideration for future studies studying the potential immunomodulatory impact of sex-hormones on the immune system.

## Evidence that the Gut-Microbiota Influences Sexual Dimorphism in Autoimmune Disease Development

Evidence from animal models of type 1 diabetes, SLE and MS–which all have a female-bias in humans–suggests that the gut-microbiota influences the sex-bias in autoimmune development. The most direct evidence comes from NOD mice, which develop a female-biased spontaneous model of type 1 diabetes. As discussed, NOD mice have a well-documented sex difference in their gut-microbiota, which influences disease development and severity. In germ-free NOD mice, the sex-bias in glucose intolerance is eradicated, suggesting a dominant role for the gut-microbiota in driving the sexual dimorphism in disease development (6). In this model, germ free mice develop diabetes more frequently than SPF mice, suggesting a protective role for the gut-microbiota (6).

SLE has one of the most pronounced sex-biases in disease development, with a female predominance ranging from 6:1 to 15:1 depending on age/study (1). In lupus-prone MRL/lpr mice, female mice, which develop more severe proteinuria, have increased levels of *Lachnospiraceae* and less *Bifidobacterium* than male MRL/lpr mice. Interestingly, female MRL/lpr mice have more *Lachnospiraceae* than female MRL control mice, whilst these differences are not observed between their male counterparts. This suggests that the increased disease severity observed in females, which is associated with high kidney damage, may be influenced by *Lachnospiraceae* (17). Validation of this theory would involve housing male and female MRL/lpr in germ-free conditions or ablating the microbiota of MRL/lpr mice with broad-spectrum antibiotics. This would allow direct comparison of lupus severity between gut-microbiota sufficient and deficient male and female mice. To our knowledge these experiments have not been performed. The immunomodulatory potential of the gut-microbiota in MRL/lpr mice is highlighted by data showing that treatment of MRL/lpr with a mixture of 5 *Lactobacillus* strains suppresses intestinal epithelium permeability (and therefore “gut leakiness”) and IgG2a production and increases IL-10 production. This leads to a reduction in lupus-associated kidney damage. In this system, treatment with *Lactobacillus* only suppresses disease in female mice and castrated male mice, but not in control males, suggesting that the impact of *Lactobacillus* on disease severity was dependent on sex-hormones (33).

In experimental autoimmune encephalitis (EAE), a mouse model of MS, treatment with high levels of the sex hormone 17-β-estradiol suppresses disease severity in C57BL/6. This is associated with alterations in the gut-microbiota, and specifically with increased abundance of *Lactobacillaceae* (34). Although the sex-bias in this model of EAE is not pronounced, the disease is known to be influenced by the microbiota, as antibiotics treatment suppresses disease development (13). This suggests an important role for the microbiota in controlling the breakdown of immunological tolerance to myelin-associated autoantigens. Models of MS that exhibit a female sex-bias, such as when EAE is induced in SJL/J mice, may offer a better system to untangle the impact of sexual dimorphism in the gut-microbiota on disease development.

Despite a pronounced sex-bias in rheumatoid arthritis development (3:1 female predominance), and the strong association between the dysbiosis and arthritis development in experimental models (9) and human patients (35), very few studies have interrogated how the gut-microbiota impacts sex-bias in experimental arthritis development. Indeed, in humanized HLA-DRB1^*^0401 mice, which develop a spontaneous female sex-bias disease (36), age- and sex-driven differences in the gut-microbiota of non-arthritic control mice are lost following arthritis development (26). Unpublished results from our laboratory suggest that under certain housing conditions, female K/BxN mice develop joint inflammation a week earlier than males, and that the disease incidence of collagen-induced arthritis in male DBA/1J is more consistent amongst different animal facilities than in female DBA/1J mice. Although we are unsure of the mechanisms underlying these colloquial observations, considering the strong influence of housing conditions on these subtle sex-differences in disease trajectory, it is tempting to speculate a dominate role for the gut-microbiota.

## Future Directions

**Figure 1:**
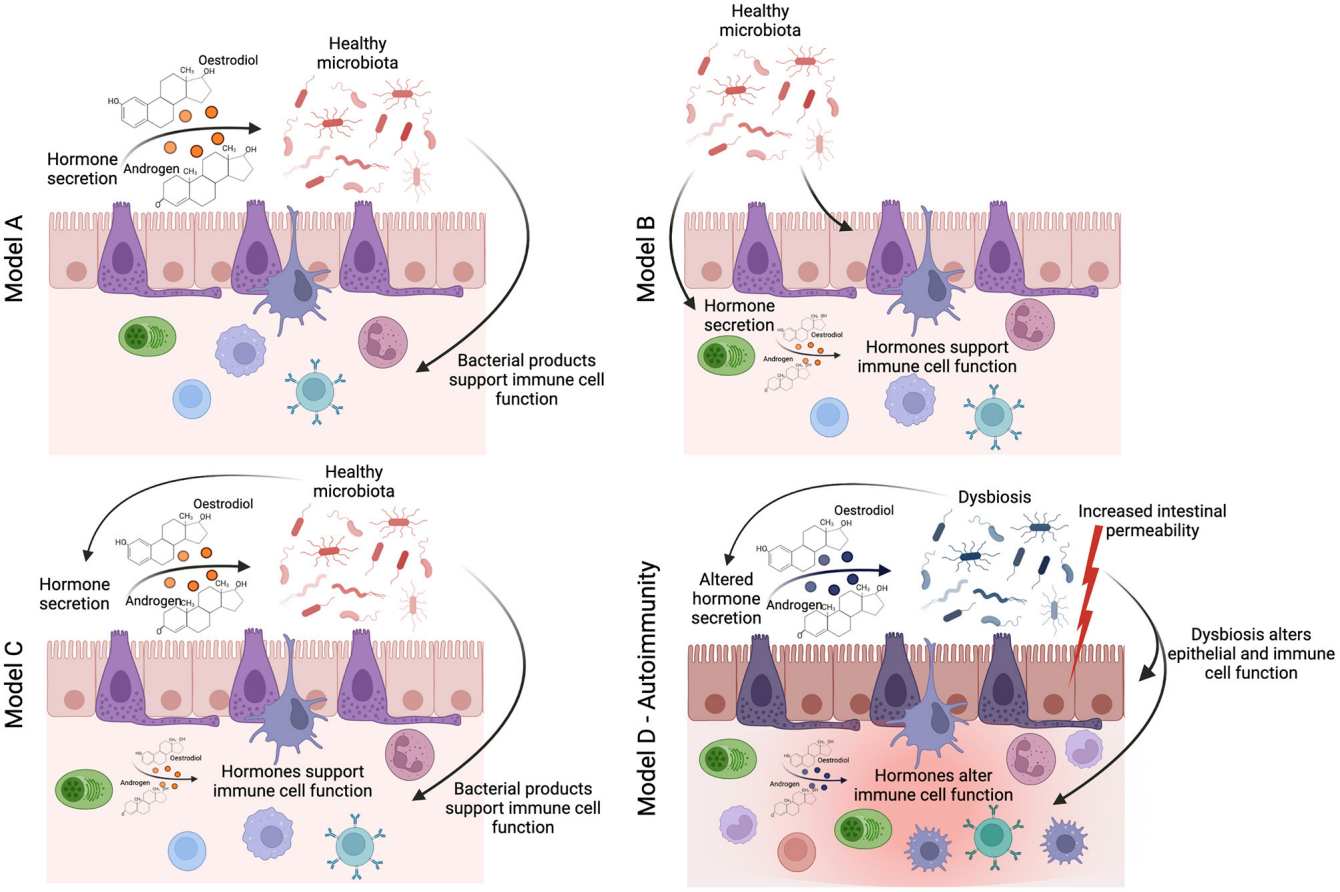
How does sexual dimorphism in the gut-microbiota influence the immune system in health and disease? The seminal study by Yurkovetskiy et al. (6) proposes three models by which the sexual dimoprhism in the gut-microbiota may influence immune system function. In linear model A, sex-hormones regulate the identity of gut-microbes (either through immune or metabolic mechanisms), and then the gut-microbes themselves activate specific immune effector mechanisms. In linear model B, gut-microbes are regulators of sex hormone metabolism, and sex-hormones are the actual effectors on immune responses. In a two-signal model C, both microbiota and hormones could contribute in an additive fashion to influence effector mechanisms (6). We would like to add an additional model for autoimmune conditions (model D), where homeostasis is lost, leading to dysbiosis, increased intestinal permeability, potential changes in the regulation of sex-hormone production, and thus altered function of the immune cell compartment and chronic inflammation.

The studies described above provide tantalizing evidence that the interplay between sex-hormones and the gut-microbiota influences the risk of developing autoimmune conditions in females. However, there remains a distinct lack of mechanistic studies that have sought to uncover the pathways by which a sexually dimorphic gut-microbiota influences chronic inflammatory states. We, and others, have shown that the gut-microbiota influences immune system function through the production of immunogenic gut-microbiota-derived metabolites and hormones such as serotonin (37). There is initial evidence that the levels of gut-derived metabolites differ between the sexes. For example, alterations in the levels of bile acids–a major group of microbiota-dependent metabolites, associated with a high-fat diet–are different in male and female mice (38). NMR-spectroscopy analysis of 24 metabolites from the gut metabolome, including short-chain fatty acids, amino acids, and other immunogenic metabolites, shows a clear difference in the gut metabolome of healthy male and female mice (39). We believe that the impact of sex on host metabolism warrants further investigation; specifically, the effect of sex-based differences in gut-microbiota-derived metabolites on immune responses is an avenue that remains unexplored. More recently, we have demonstrated that other features of gut health, such as intestinal permeability, correlate with the onset and progression of autoimmune inflammation (40). It has been suggested that female mice have higher baseline intestinal permeability (41), and the expression of genes involved in mucus biosynthesis in the ileum–which plays an essential role in supporting a healthy gut barrier–are differentially regulated in old male and female mice (42). Moving forwards from three potential models proposed by Yurkovetskiy and colleagues of how the gut-microbiota may influences sex-hormones production or vice versa (6): we propose that future studies should consider the diverse impact of the gut-microbiota on host metabolism and barrier function, allowing the identification of mechanisms in which sexual dimorphism in the gut-microbiota influences chronic inflammatory states (Figure 1). This is essential to identify novel “druggable” pathways for the prevention or suppression of sex-biased inflammatory disease processes.

## Conclusions

Although the impact of sex and the gut-microbiota on autoimmunity are clearly defined, the understanding of how these two risk factors may impact one another is ill-defined. In this review, we have summarized current literature from animal models that suggest that the gut-microbiota differs between the sexes in the steady state and in inflammatory conditions. However, this relationship is complex, and is influenced by other factors including diet, housing and genotype. As translation of animal research into humans is the central tenet for the use of experimental models, these data underline the importance of collecting in-depth demographic information–such as age, ethnicity, body mass index, diet and medications–when comparing sex-differences in the human microbiota in healthy individuals and individuals with autoimmune conditions. Initial studies of the human microbiota have reported that there is sexual dimorphism in the gut-microbiota (43), and that there is a potential correlation between the diversity and richness of the gut-microbiota and urinary sex-hormones, namely estrogen, levels (44). In future studies, considering the impact of genotype on differences in the gut-microbiota between male and female mice, where possible, it would be informative to collect genotype data alongside bacterial sequencing data in large scale population studies of the human gut-microbiota. These complex large-scale studies would be critical in providing new insights into the potential directionality between sex, environment, and genetic risk when considering biological pathways contributing to autoimmune disease pathophysiology.

We have also highlighted the intimate connection between the gut-microbiota and sex-hormones, and in particular testosterone. Modulation of the gut-microbiota may represent a potential, less invasive, treatment strategy than injection of high levels of hormones, specifically in conditions where levels of sex-hormones are thought to influence disease development–such as reduction in testosterone levels in SLE patients (45, 46)–or in conditions where hormone therapy has been suggested as a potential treatment strategy–such as androgen treatment in MS (47) and in SLE (48).

Finally, we emphasize the need for mechanistic experiments that interrogate how sexual dimorphism in the gut-microbiota alters immune responses in such a way that renders them pathogenic in autoimmune conditions. Based on our previous research, we suggest that investigating how sex-differences in the gut-microbiota change immunogenic gut-derived metabolites or gut-barrier function may provide exciting new research opportunities. The reports summarized in this review show that the study of the direct and indirect pathways by which sex influences immune responses, and thus autoimmune development, is a field in its infancy. Altogether, these data highlight the need to disaggregate all aspects of medical research–including the study of the microbiota–by sex and gender, especially when considering the biological pathways that underlie the development of autoimmunity.

## Author Contributions

ER conceptualized the review and wrote the manuscript. NG critically reviewed the manuscript. DM reviewed the manuscript and made figures and tables. All authors contributed to the article and approved the submitted version.

## Funding

ER and NG are supported by Medical Research Foundation Fellowship awarded to ER (MRF- 057-0001-RG-ROSS-C0797). ER is also supported by a Versus Arthritis Center for Excellence grant awarded to Professor L. Wedderburn (21593). DM is supported by a Versus Arthritis project grant to Professor C. Mauri (21786). This work is supported by an MRC project grant awarded to Professor C. Mauri, ER and Dr. P. Blair (MR/T000910/1). Figure was created with BioRender.com.

## Conflict of Interest

The authors declare that the research was conducted in the absence of any commercial or financial relationships that could be construed as a potential conflict of interest.

## Publisher's Note

All claims expressed in this article are solely those of the authors and do not necessarily represent those of their affiliated organizations, or those of the publisher, the editors and the reviewers. Any product that may be evaluated in this article, or claim that may be made by its manufacturer, is not guaranteed or endorsed by the publisher.
